# Effect of water soluble humic acid applied to potato foliage on plant growth, photosynthesis characteristics and fresh tuber yield under different water deficits

**DOI:** 10.1038/s41598-020-63925-5

**Published:** 2020-05-12

**Authors:** Yang Man-hong, Zhang Lei, Xu Sheng-tao, Neil B. McLaughlin, Liu Jing-hui

**Affiliations:** 1 Oat Scientific and Technical Innovation Team, Inner Mongolia Agricultural University, Hohhot, Inner Mongolia 010019 China; 2 Institute of Economic Crops, Yunnan Academy of Agricultural Sciences, Kunming, Yunnan 650205 China; 3 Agricultural Environment and Resources Institute, Yunnan Academy of Agricultural Sciences, Kunming, Yunnan 650205 China; 4 Ottawa Research and Development Centre, Agriculture and Agri-Food Canada, Ottawa, ON K1A 0C6 Canada

**Keywords:** Photosynthesis, Plant development, Plant physiology, Plant stress responses

## Abstract

Water scarcity is the main limiting factor in agricultural crop production in arid and semi-arid areas in northern China. Humic acid could improve the plant resistance to mitigate the abiotic drought damages, which is a potential strategy to improve the crop production in these regions. An experiment to investigate the effect of water soluble humic acid on plant growth, photosynthesis characteristics and fresh tuber yield of potato under different water deficits was carried out under greenhouse conditions in 2014 and 2015. Treatments included foliar application of fresh water (FW), humic acid diluted with water 500 times (HA) and control (CK), and the water deficits included 45%, 60% and 75% of the field water holding capacity. The HA treatment showed highly significant (*P* ≤ 0.01) effect on dry biomass, root/shoot ratio and photosynthesis parameters, improved the dry biomass above ground (DM-AG) by 14.12–36.63%, 11.62–36.26% and 7.85–20.85% over the whole growing season at water deficits of 45%, 60% and 75% of the field water holding capacity respectively in 2014 and 2015; decreased the root/shoot (R/S) ratio in the early growing season and increased the R/S ratio in the later growing season; showed an improved effect on leaf soil plant analysis development (SPAD), photosynthesis rate (Pn) and stomatal conductance (Gs) and decreased transpiration rate (Tr) and intercellular CO_2_ concentration (Ci) compared with the control. HA usually showed a better effect on photosynthesis parameters in 60% of the field water holding capacity than 45% and 75% except on Pn. Compared with control, HA increased fresh tuber yield by 34.47–63.48%, 35.95–37.28% and 23.37–27.15% at 45%, 60% and 75% of the field water holding capacity respectively. HA enhanced the potato plant growth, and improved photosynthesis parameters and fresh tuber yield under different water deficits under green house conditions, and represents an opportunity to improve crop production and sustainability of agriculture in arid and semiarid regions.

## Introduction

Water scarcity is an abiotic stresses and directly affects photosynthetic pigments, respiration, leaf area index, root growth, nutrient metabolism and hormonal balance, which in turn affect the plant growth and development^1–4^. Water deficit caused by drought is becoming the determinant factor in arid and semiarid regions^5^, and has resulted in yield and economic loss^6^. Previous studies have shown that reduced tillage, and crop breeding for drought resistance are potential strategies to mitigate the drought hazard^7–12^. Humic acid (HA) applied to the plant foliage is an effective way to improve the plant resistance to abiotic drought stresses by influencing the antioxidant defense mechanisms^13,14^. HA has been shown increase the nutrient uptake and utilization, and improve crop yield^15,16^.

Photosynthesis characteristics, such as leaf soil plant analysis development (SPAD) value, net photosynthesis rate (Pn), stomatal conductance (Gs), transpiration rate (Tr) and intercellular CO_2_ concentration (Ci), play a key role in crop productivity, and are closely related to the yield^17,18^. Nevertheless, these photosynthesis parameters are sensitive to unfavorable environmental conditions such as drought, salinity and heat^19–21^, and plant photosynthesis activity shows a decreasing trend with the increasing water stress^22^. HA mixed with water-absorbing agent increased photosynthetic parameters and tuber nutritional quality in a semi-arid region^2^, and most of the research used HA as the soil amendments, and focus on the effect of HA on soil water content to promote the plant growth, but there was less result for HA under the different gradient of water deficits. Some other result showed that HA could improve the plant resistance of corn and wheat by influencing the antioxidant defense mechanisms^13,14^, and indirectly increased the plant photosynthesis characteristics under unfavorable conditions. However, there is little information on the response of potato to HA under different soil water contents.

The objective of this study was to determine the effect of HA on photosynthesis and tuber yield in different drought conditions, and the above and under ground biomass and tuber yield were measured for the effect of HA on the biomass accumulation, leaf soil plant analysis development (SPAD) value, net photosynthesis rate (Pn), stomatal conductance (Gs), transpiration rate (Tr) and intercellular CO_2_ concentration (Ci) were measured for the effect of HA on the photosynthetic characteristics.

## Results

### ANOVA of measurements

The ANOVA of different measurements is shown in Table 1. HA treatments (T), water deficits (W) and growth stage (S) all showed highly significant (*P* ≤ 0.01) effect on dry biomass, root/shoot ratio and photosynthesis parameters in 2014 and 2015. The interaction between HA treatments (T) and growth stage (S) showed significant (*P* ≤ 0.05) effect on DM-AG, DM-UG, R/S, Gs, Ci and Tr, but showed no significant (*P* > 0.05) effect on SPAD and Pn. The interaction between water deficits (W) and growth stage (S) showed highly significant (*P* ≤ 0.01) effect on all parameters except SPAD where the effect was not significant(*P* > 0.05). The interaction between HA treatments (T) and water deficits (W) showed highly significant (*P* ≤ 0.01) effect on DM-UG, Tr and Gs, showed on no significant(*P* > 0.05) effect on DM-AG, R/S, SPAD, Pn and Ci. The interaction among HA treatments (T), water deficits (W) and growth stage (S) showed no significant (*P* > 0.05) effect on all parameters except the DM-UG with highly significant (*P* ≤ 0.01) effect.

**Table 1:** ANOVA of effect of treatments (T), water gradients (W) and growth stage (S) on dry biomass, root/shoot ratio and photosynthesis parameters in 2014 and 2015.

Factor	DF	DM-AG	DM-UG	R/S	SPAD	Pn	Tr	Gs	Ci
T	2	***	***	**	***	***	***	***	***
W	2	***	***	***	***	***	***	***	***
S	2	***	***	***	***	***	***	***	***
T*S	4	***	***	***	NS	NS	***	***	*
S*W	4	***	***	***	NS	***	***	***	**
W*T	4	NS	***	NS	NS	NS	**	**	NS
W*S*T	8	NS	***	NS	NS	NS	NS	NS	NS

Significance: *P < 0.05; **P < 0.01; ***P < 0.001; NS, not significant (P > 0.05). DF, degrees of freedom; DB-AG, dry biomass above ground; DB-UG, dry biomass underground; R/S, crop root/shoot ratio; SPAD, leaf soil plant analysis development value; Pn, net photosynthesis rate; Gs, stomatal conductance; Ci, intercellular CO2 concentration; Tr, transpiration rate.

### Root/shoot ratio

**Figure 1:**
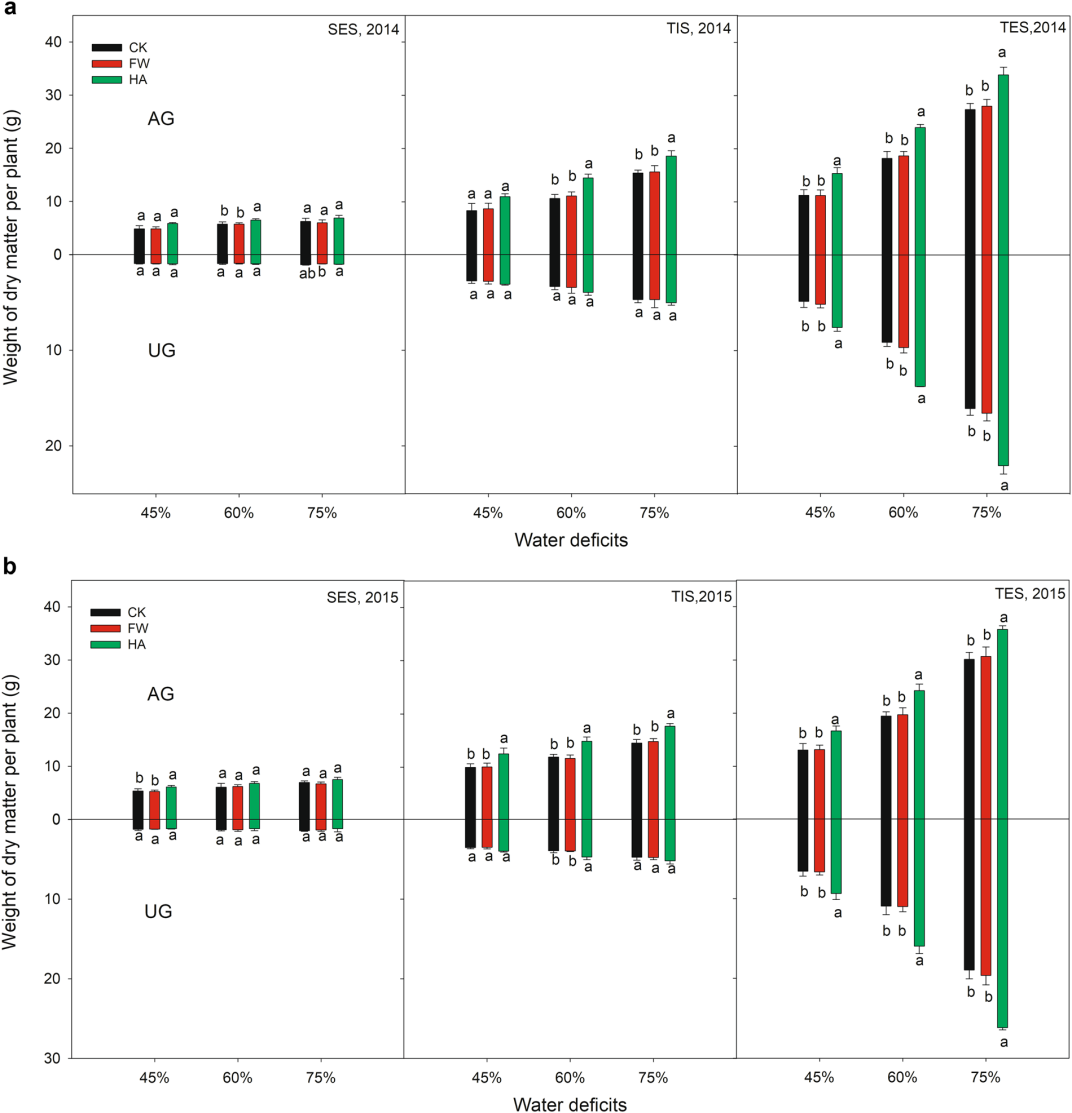
(**a**) The variation of root and shoot dry biomass with water-soluble humic acid at different water gradients in 2015. Treatment code: CK, non-application control; FW, application of fresh water; HA, application of humic acid with dilute 500 times. Small bar shows standard deviation. Columns within the same year and stage and with the same letters are not significantly different at *P* = 0.05 according to a protected LSD test.

The response of DM-AG and DM-UG of potato with different treatments at different growth periods and different water deficits are presented in Fig. 1a,b. HA improved the DM-AG by 14.12–36.63%, 11.62–36.26% and 7.85–20.85% over the whole growing season at 45%, 60% and 75% of the field water holding capacity respectively in 2014 and 2015. HA always significantly (*P* ≤ 0.05) improved DM-AG of potato compared to the control at TIS and TES under different water deficits in 2014 and 2015. Meanwhile, HA improved the DM-UG by (−)6.80–56.58%, (−)11.99–50.61% and (−)16.94–38.18% over the whole growing season at 45%, 60% and 75% of the field water holding capacity respectively in 2014 and 2015. HA always significantly (*P* ≤ 0.05) improved DM-UG of potato over the control in the later growing season (TES) under different water deficits in 2014 and 2015, but showed a significant (*P* ≤ 0.05) reduction effect compared with the control in the early growing season (SES).

**Figure 2:**
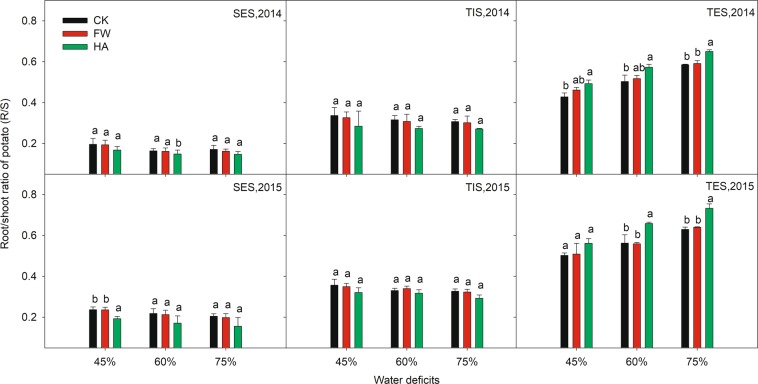
The variation of root/shoot ratio with water-soluble humic acid at different water gradients in 2014. Treatment code: CK, non-application control; FW, application of fresh water; HA, application of humic acid with dilute 500 times. Small bar shows standard deviation. Columns within the same year and stage and with the same letters are not significantly different at *P* = 0.05 according to a protected LSD test.

The variation of R/S ratio of potato with HA at different water deficits are plotted in Fig. 2. HA decreased the R/S ratio in the early growing season (SES and TIS) by 14.10–19.04%, 7.55–16.71%, 11.84–17.79% at 45%, 60% and 75% of the field water holding capacity respectively in 2014 and 2015. However, HA increased the R/S ratio in the later growing season (TES) by 11.82–15.03%, 13.92–17.13% and 11.00–16.48% at 45%, 60% and 75% of the field water holding capacity respectively in 2014 and 2015, and HA always showed a significant (*P* ≤ 0.05) difference compared with the control at different water deficits in 2014 and 2015.

### Photosynthesis parameters

**Figure 3:**
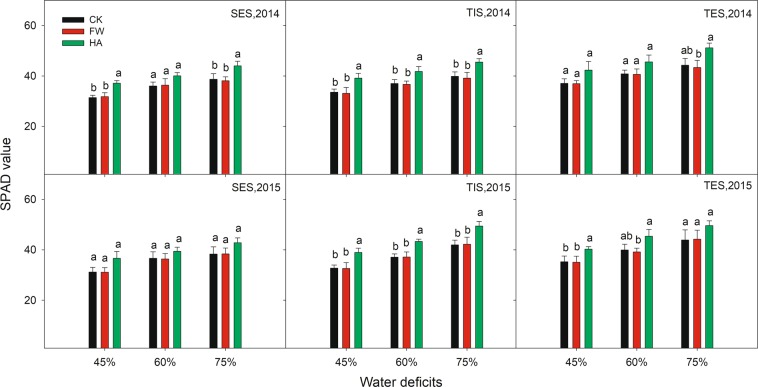
The variation of leaf SPAD value with water-soluble humic acid at different water gradients in 2014. Treatment code: CK, non-application control; FW, application of fresh water; HA, application of humic acid with dilute 500 times. Small bar shows standard deviation. Columns within the same year and stage and with the same letters are not significantly different at *P* = 0.05 according to a protected LSD test.

**Figure 4:**
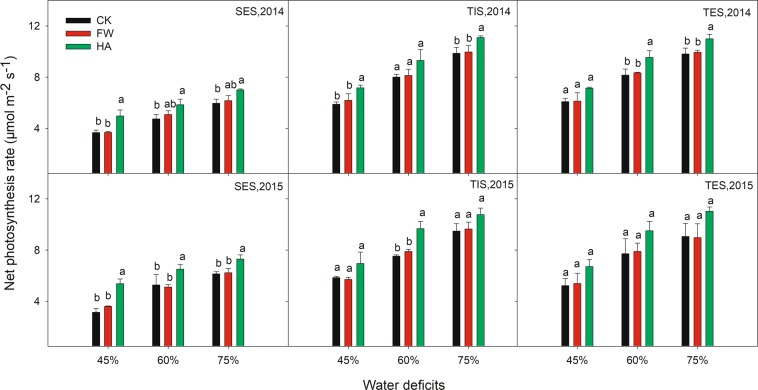
The variation of net photosynthesis rate with water-soluble humic acid at different water gradients in 2014. Treatment code: CK, non-application control; FW, application of fresh water; HA, application of humic acid with dilute 500 times. Small bar shows standard deviation. Columns within the same year and stage and with the same letters are not significantly different at *P* = 0.05 according to a protected LSD test.

**Figure 5:**
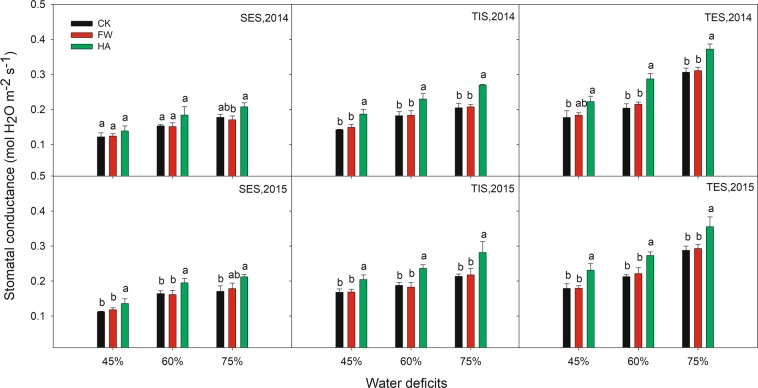
The variation of stomatal conductance with water-soluble humic acid at different water gradients in 2014. Treatment code: CK, non-application control; FW, application of fresh water; HA, application of humic acid with dilute 500 times. Small bar shows standard deviation. Columns within the same year and stage and with the same letters are not significantly different at *P* = 0.05 according to a protected LSD test.

**Figure 6:**
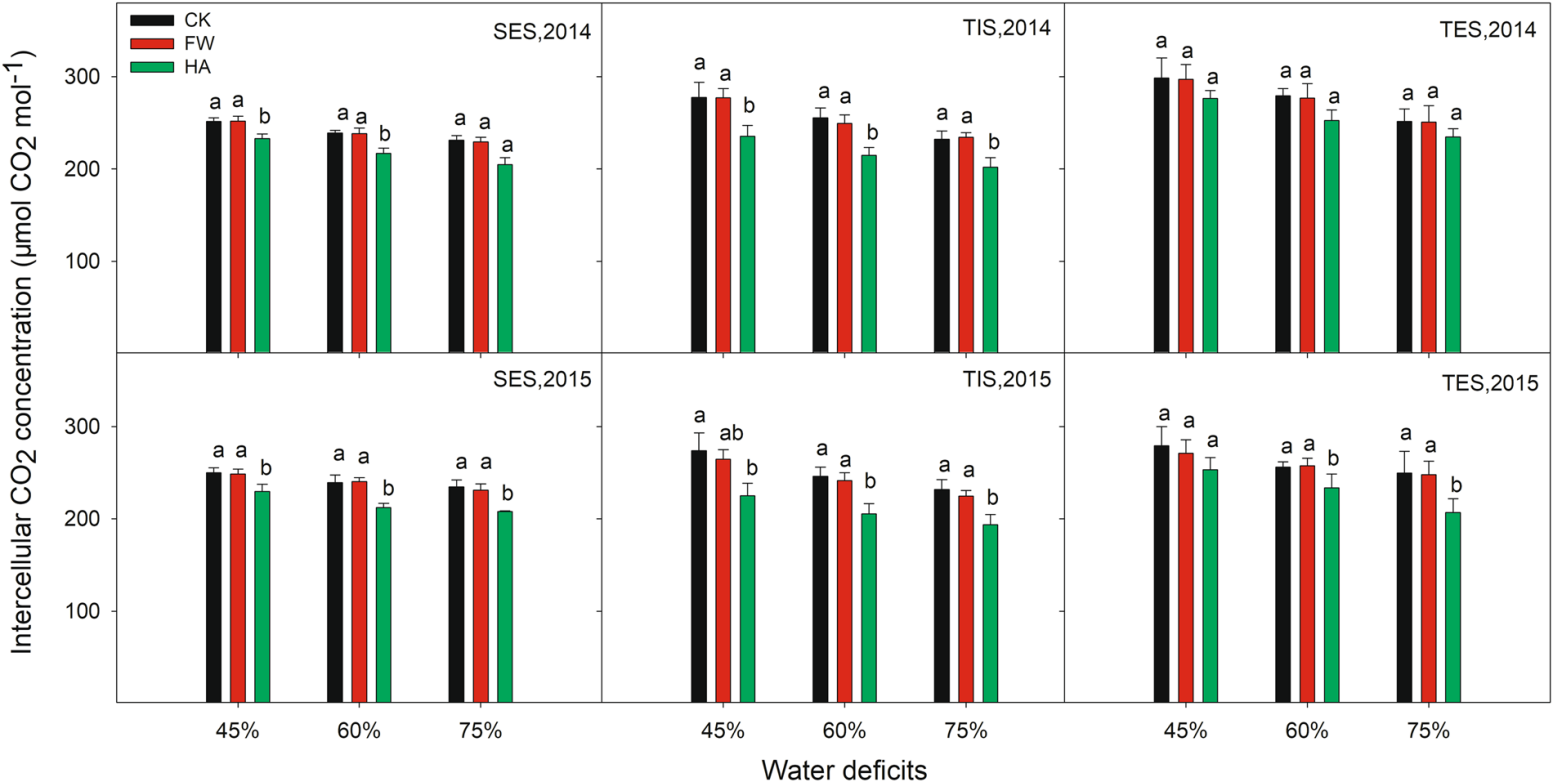
The variation of intercellular CO_2_ concentration with water-soluble humic acid at different water gradients in 2014. Treatment code: CK, non-application control; FW, application of fresh water; HA, application of humic acid with dilute 500 times. Small bar shows standard deviation. Columns within the same year and stage and with the same letters are not significantly different at *P* = 0.05 according to a protected LSD test.

**Figure 7:**
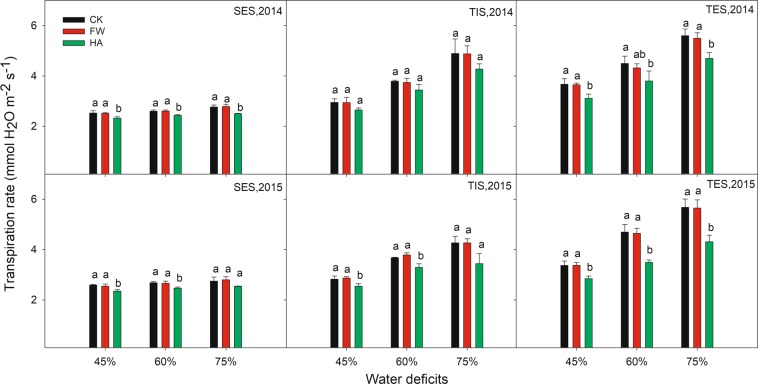
The variation of transpiration rate with water-soluble humic acid at different water gradients in 2014. Treatment code: CK, non-application control; FW, application of fresh water; HA, application of humic acid with dilute 500 times. Small bar shows standard deviation. Columns within the same year and stage and with the same letters are not significantly different at *P* = 0.05 according to a protected LSD test.

HA showed an improvement effect on SPAD, Pn and Gs compared with the control at different water deficits in 2014 and 2015 (Figs. 3–5 respectively). HA increased SPAD by 11.82–15.03%, 13.92–17.13% and 11.00–16.48% respectively, Pn by 17.31–70.97%, 16.22–28.45% and 11.96–21.71% respectively, Gs by 13.68–31.74%, 18.77–40.83% and 16.78–31.55% respectively at 45%, 60% and 75% of the field water holding capacity in 2014 and 2015. Nevertheless, HA decreased Ci and Tr compared with the control at different water deficits in 2014 and 2015 (Figs. 6 and 7 respectively). HA reduced Ci by 7.27–17.86%, 9.19–16.49% and 6.55–17.22%, Tr by 7.82–15.56%, 6.37–25.56% and 7.45–24.03% respectively at 45%, 60% and 75% of the field water holding capacity in 2014 and 2015. There was a similar trend of HA effect on the photosynthesis measurements at different growth stages in different water deficits in 2014 and 2015. HA usually showed a greater effect on photosynthesis parameters at 60% of the field water holding capacity than at 45% or 75% except on Pn.

### Yield

Fresh tuber yield for different treatments at different water deficits in 2014 and 2015 is presented in Tables 2 and 3. Compared with control, HA increased fresh tuber yield by 34.47–63.48%, 35.95–37.28% and 23.37–27.15% at 45%, 60% and 75% of the field water holding capacity respectively in 2014 and 2015. Simultaneously, HA always showed a significant (*P* ≤ 0.05) improvement over CK at different water d in 2014 and 2015, and the effect of HA showed an improvement trend on the fresh tuber yield with decreasing soil water content.

**Table 2:** ANOVA of effect of treatments (T), water gradients (W), year (Y) and stage (S) on dry biomass, root/shoot ratio and photosynthesis parameters in 2014 and 2015.

Factor	DF	DM-AG	DM-UG	R/S	SPAD	Pn	Tr	Gs	Ci
T	2	***	***	NS	***	***	***	***	***
W	2	***	***	***	***	***	***	***	***
W*T	4	NS	***	NS	NS	NS	**	**	NS
Y	1	***	***	***	NS	*	***	NS	***
Y*T	2	NS	NS	NS	NS	*	*	NS	NS
Y*W	2	NS	NS	NS	NS	NS	**	NS	NS
Y*W*T	4	NS	NS	NS	NS	NS	NS	NS	NS
S	2	***	***	***	***	***	***	***	***
S*T	4	***	***	***	NS	NS	***	***	*
W*S	4	***	***	***	NS	***	***	***	**
W*S*T	8	NS	***	NS	NS	NS	NS	NS	NS
Y*S	2	***	***	***	*	**	***	**	***
Y*S*T	4	NS	NS	*	NS	NS	NS	NS	NS
Y*W*S	4	***	***	NS	NS	NS	***	*	NS
Y*W*S*T	8	NS	NS	NS	NS	NS	NS	NS	NS

Significance: *P < 0.05; **P < 0.01; ***P < 0.001; NS, not significant (P > 0.05). DF, degrees of freedom; DB-AG, dry biomass above ground; DB-UG, dry biomass underground; R/S, crop root/shoot ratio; SPAD, leaf soil plant analysis development value; Pn, net photosynthesis rate; Gs, stomatal conductance; Ci, intercellular CO2 concentration; Tr, transpiration rate.

**Table 3:** Fresh tuber yield for different treatments at different water gradients in 2014 and 2015.

Water gradients	Treatments	Fresh tuber yield (g plant^−1^)	
		2014	2015
45%	FW	5.67(0.69) b	7.45(0.87) b
	CK	5.12(0.97) b	7.41(0.90) b
	HA	8.37(0.99) a	9.96(0.58) a
60%	FW	11.14(1.50) b	12.43(0.73) b
	CK	11.41(0.74) b	11.78(1.45) b
	HA	15.67(1.69) a	16.02(0.90) a
75%	FW	61.58(3.76) b	70.52(6.10) b
	CK	60.15(2.12) b	72.45(5.29) b
	HA	76.48(4.98) a	89.39(4.81) a

Means in the same column and water gradient, and followed by the same letter are not significantly different (P > 0.05) according to a protected LSD test. Numbers in parentheses are standard deviation. Treatment code: CK, non-application control; FW, application of fresh water; HA, application of humic acid with dilute 500 times.

## Discussion

Our data showed that HA increased the DM-AG, fresh tuber yield and DM-UG at TIS and TES in different water deficits in 2014 and 2015 (Fig. 1a,b, Table 1). HA could promote the potato growth from the result fresh tuber yield, DM-AG and DM-UG in the later growing season, which is consistent with previous results for corn and potato yield^7,24^. Meanwhile, the effect of HA showed an increasing trend on the fresh tuber yield with increasing the water deficit demonstrating that crop plant growth was significantly affected by the water condition^25,26^. The highest increase in tuber yield with the HA was obtained at 75% of the field water holding capacity, which indicates HA is more effective with adequate water condition than water deficit condition. Nevertheless, HA showed a decreased effect on the R/S ratio in the early growing season (SES and TIS) at different water deficits in 2014 and 2015, which was related to the characteristics of HA and the application method. HA could mitigate the damage of water deficit^13,14^. Spraying HA on the potato plant leaves directly and quickly influenced the growth of the above ground parts of the potato plant, but there was a certain lag on the growth of below ground parts of the potato plant. HA increased the R/S ratio in the later growing season (TES), which indicated that HA promoted the potato growth, and increased the nutrients were transported to belowground at the same time in this period which in turn improved fresh tuber yield.

HA improved the SPAD, Pn and Gs and decreased the Ci and Tr at different water deficits in 2014 and 2015 (Figs. 3–7). This indicated that HA could improve the potato resistance to, and reduce the damage from drought stress by adjusting the physiological and biochemical activities^27,28^. This is in accordance with previous research which found HA could promote plant growth and development by improving the photosynthesis parameters for chrysanthemums^29^ and peas^30^. Moreover, this situation would in turn influence the plant’s nutrient utilization and dry matter accumulation under unfavorable conditions. Our result showed that DM-AG, fresh tuber yield and DM-UG at later growing season (TES) was improved by the HA under different water deficits in 2014 and 2015, which is agreement with the improvement in photosynthetic activities^17,18^ HA usually showed a better effect on photosynthesis parameters in 60% of the field water holding capacity than at 45% or 75% except on Pn. Simultaneously, Tr was reduced by HA, indicating that HA can improve the water use efficiency, which is beneficial for the crop production in the arid and semiarid areas where water scarcity is the key limitation in agriculture crop production^31^. These results all demonstrate that HA could contribute to the sustainable agriculture in the arid and semiarid regions in the Northern China.

In this experiment, we had different water stresses, and it is possible that the water in the HA treatment may have affected, the potato plant growth and development. Our result showed that there were no significant (*P* > 0.05) differences between the fresh water treatment and control, but HA usually showed a significant (*P* ≤ 0.05) effect on different measurements under different water deficit; this indicates that the water in the HA had no effect and that HA is the main factor which influences the potato plant growth and photosynthetic activities in the HA treatment. This is in agreement with previous research that HA could enhance the crop resistance to water stress, and improve the nutrient uptake and utilization, and thus increase crop yield under unfavorable soil water conditions^14–16^. There are many studies that focused on applying HA into the soil, which improved the soil physical, chemical and biological properties, especially for the soil in the arid and semiarid areas^32–35^. Nevertheless, we need more information on the effect of HA on the plant physiological and metabolic processes under unfavorable stresses, both in the green house and field to achieve a more complete understanding for the effect of HA on plant growth and development.

## Conclusion

Our study indicates that foliar application of HA can enhance the potato plant growth, and improves photosynthesis parameters and fresh tuber yield under different water deficits under green house conditions, which represents an opportunity to improve crop production and sustainability agriculture in the arid and semiarid regions. Further research of the effect of HA on physiological and metabolic processes under unfavorable stresses, is required for a more complete understanding to provide a basis of the development of management strategies for improvement crop production in the arid and semiarid areas.

## Materials and Methods

A green house trial was conducted in 2014 and 2015, to determine the effect of water-soluble HA applied to the foliage under different drought conditions on photosynthesis characteristics of a potato crop including leaf SPAD value, Pn, Ci, Tr, dry biomass, root/shoot ratio and potato tuber yield.

### Experiment site

The green house trial was conducted at Oat Scientific and Technical Innovation Team, Inner Mongolia Agricultural University, Hohhot, Inner Mongolia, China in 2014 and 2015. The greenhouse was maintained at 25 °C day and 18 °C, reduced tillage, and crop breeding for drought with natural light; on cloudy and rainy days, natural light was supplemented with 4000 lx artificial light.

### Experiment design

The green house trail was a randomized complete block factorial experimental design with treatments of three foliar treatments (HA, water and control), three growth stages (40, 60 and 80 days after planting) and three water deficits of 45, 60 and 75% of field capacity representing arid, semi-arid and normal conditions, and with three replicates. Potato plants were grown in 20 cm dia. x 25 cm high pots, one plant per pot. Each pot was filled with 3.5 kg soil mixed with 0.2 kg peat, and the soil was same for the two years’ experiment. The soil was sandy loam, with pH of 7.82, and containing (g kg^−1^) 29.60 organic carbon, 0.20 alkaline nitrogen, 0.0040 available phosphorus, and 0.21 available potassium. Compound granular fertilizer (17–6–23) was applied and 2 cm dia. virus free micro-tubers were planted, at 8 cm depth, one tuber per pot on April 19^th^, 2014 and April 24^th^, 2015; there were 20 pots for each replicate. The potato cultivar was Atlantic, which is commonly grown in arid and semi-arid areas in Inner Mongolia. Soil chemical properties are showed in Table 4.

**Table 4:** Soil chemical properties of the green house experiments.

Measurements	Alkaline nitrogen (mg·kg^−1^)	Available phosphorus (mg·kg^−1^)	Available potassium (mg·kg^−1^)	Organic carbon (g·kg^−1^)	pH value
Values	196.50	4.40	210.40	29.6	7.82

The water deficits were maintained by weighing the pots each week, and adding water to maintain the water deficits of 45, 60 and 75% of field capacity; potato biomass was estimated at each growth stage and the amount of water added was adjusted to reflect the increase in pot weight due to potato biomass.

The treatments were application of fresh water (FW), application of HA diluted 500 times (HA) and control (CK); the HA and fresh water were applied by spraying on the potato foliage at seedling stage (SES, 40 d after planting), tuber initiation stage (TIS, 60 d after planting) and tuber expansion stage (TES, 80 d after planting). The HA was diluted at 500 times for spraying, and application rates for FW and HA were 50 ml per plant at the above three growth stages; this corresponds to a field application rate of 4.5 L ha^−1^. HA was made by Yongye Group Co., Ltd., Hohhot, Inner Mongolia, China; the free HA purity was up to 95%.

### Greenhouse and laboratory measurements

In each plot, three randomly selected plants were dug one week after each application of HA and fresh water at SES, TIS and TES for determining fresh and dry plant mass. These samples were divided into underground (including tubers) and above-ground parts, and oven-dried at 105 °C for 30 min and then at 80 °C until constant weight for dry mass. Root/shoot (R/S) ratio was calculated on a dry matter basis for the above and below ground parts of the plant.

Net photosynthesis rate (Pn), intercellular CO_2_ concentration (Ci) and transpiration rate (Tr) were measured at SES, TIS and TES with a portable photosynthesis system (LI-6400, Li-Cor Inc., Lincoln NE, USA) equipped with a standard 18 cm^3^ prismatic leaf chamber. Determinations were conducted under a standard constant air flow rate of 500 μmol s^−1^ with the natural light on a sunny day; measurement time was between 09:00 and 11:00 when the air temperature was 24 ± 2 °C. Measurements were made on three randomly selected mature leaves; mature leaves are known to exhibit higher stability and response of photosynthesis rates to external factors^23^.

Leaf soil plant analysis development (SPAD) values were measured at SES, TIS and TES with a chlorophyll meter (SPAD-502 Plus, Konica Minolta, Tokyo, Japan). Three randomly selected leaves of similar size, maturity and location on potato were used for SPAD values.

Yield was determined by harvesting three plants at maturity.

### Data analysis

Analysis of variance (ANOVA) was performed using SAS Version 9.3 (SAS Institute Inc., Cary, NC, USA). Tests of significance used the least significant difference (LSD) at *P* ≤ 0.05. Mean values are reported in the tables and figures.
